# The complete chloroplast genome of *Camellia osmantha*, an edible oil Camellia

**DOI:** 10.1080/23802359.2021.1987169

**Published:** 2021-10-12

**Authors:** Yanju Liu, Yufen Xu, Xiaocheng Jia

**Affiliations:** Hainan Key Laboratory of Tropical Oil Crops Biology/Coconut Research Institute, Chinese Academy of Tropical Agricultural Sciences, Wenchang, Hainan, China

**Keywords:** *Camellia osmantha*, chloroplast genome, phylogenetic analysis

## Abstract

*Camellia osmantha* is a new species of the *Camellia* genus discovered in Nanning, Guangxi, China, in 2012. It can be used as an excellent woody oil crop. There is little related research on this species in China and abroad, and its genome information is still lacking. In this study, the complete chloroplast genome sequence of *C. osmantha* was first reported (GenBank number: MZ128138). The whole chloroplast genome is 156,981 bp in length with a GC content of 37.28%, and it is composed of a large single copy (LSC) region of 86,647 bp, a small single copy (SSC) region of 18,284 bp, and a pair of inverted repeat (IR) regions of 26,025 bp each. The genome contains a total of 135 functional genes, including 37 transfer RNA genes, 90 protein-coding genes, and 8 ribosomal RNA genes. The maximum likelihood analysis based on 21 chloroplast genomes showed that *C. osmantha* and *C. oleifera* (MF541730.2) were the most closely related.

*Camellia osmantha* is a new species discovered in Nanning, Guangxi, China, in 2012. It can be used as an excellent woody oil crop with the characteristics of large fruit quantity, early maturity, and high seed yield (Ouyang et al. 2020). Compared with ordinary *Camellia oleifera*, this new species shows stronger growth potential and stress resistance (Jiang et al. 2016). It is distributed in the tropics and the northern edge of the tropics and has physiological and ecological characteristics that make it well adapted to the tropical climate. The fruit of *C. osmantha* can be squeezed into oil and has high economic value, and the oil is rich in several fatty acids, including stearic, linoleic, and palmitic acids (Xia et al. 2014). Simultaneously, *C. osmantha* can be used as an ornamental tree species with a beautiful tree shape and fragrant flowers (Ma et al. 2012). There is little research on this species in China and abroad, and its genome information is still lacking. Therefore, we assembled and annotated the complete chloroplast (cp) genome (NCBI accession number: MZ128138) of *Camellia osmantha* by high-throughput sequencing, which can provide a theoretical basis for phylogenetics,comparative studies and chloroplast engineering of *C. osmantha*.

The fresh leaves of *C. osmantha* from the Coconut Research Institute of CATAS (Hainan, China; Coordinates: 19°32′4.92″'N, 110°45′47.29″'E) were used as research materials, and the certificate specimen (XH1) is stored in the Camellia Research Center, Coconut Research Institute of CATAS. Genomic DNA was extracted by Takara DNA extraction kit (Takara, Dalian, China); library construction and sequencing were performed on the Illumina HiSeq 2500 second-generation platform; and chloroplast assembly software GetOrganelle (Jin et al. 2020) was used for genome assembly. We used the online annotation software Geseq (Tillich et al. 2017) and CpGAVAS (Liu et al. 2012) to assemble the cp genome annotation, *Camellia pubicosta* (NC_024662.1) was used as reference genome. After proofreading and correction, the complete cp genome was submitted to GenBank (NCBI accession number: MZ128138).

The *C. osmantha* cp genome is a circular molecule with a total length of 156,981 bp, containing a large single copy (LSC) region of 86,647 bp, a small single copy (SSC) region of 18,284 bp, and a pair of inverted repeat (IR) regions of 26,025 bp each. The total GC content is 37.28%. The corresponding GC contents in the LSC, SSC, and IR regions are 35.3, 30.5, and 43.0%, respectively. The cp genome has 135 functional genes, including 37 transfer RNA genes, 90 protein-coding genes, and 8 ribosomal RNA genes.

The complete cp genomes of *C. osmantha* and 17 other *Camellia* species were used for phylogenetic analysis, and RAxML 8.2.12 (Stamatakis 2014) was used to construct a maximum likelihood (ML) tree in GTRGAMMA model, with three closely related *Camellia* species as the outgroups. As shown in Figure 1, in this study, all the *Camellia* plants gathered into an independent branch, and the relationship between *C. osmantha* and *C. oleifera* (MF541730.2) was found to be the closest.

**Figure 1.**
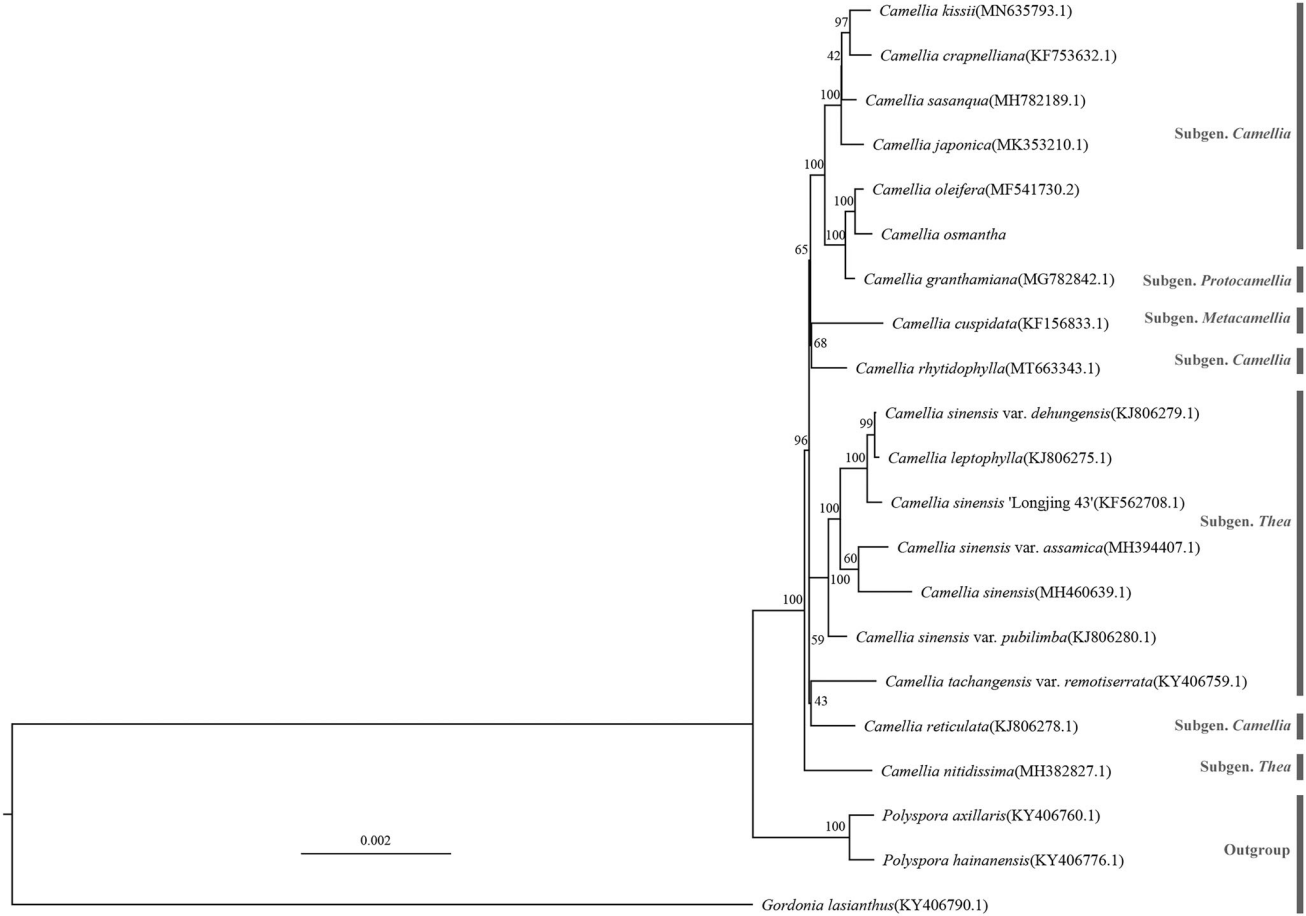
A phylogenetic tree of Camellia based on 21 complete chloroplast genome sequences. The bootstrap values from 1000 replicates are listed on each node.

## Data Availability

The genome sequence data that support the findings of this study are openly available in GenBank of NCBI at (https://www.ncbi.nlm.nih.gov/) under the accession No. MZ128138. The associated BioProject, Bio-Sample, and SRA numbers are PRJNA725044, SAMN18928057, and SRR14469701 respectively.
